# Digital Exclusion and Sleep Problems in Middle-Aged and Older Adults: A Multi-Cohort Cross-National Study

**DOI:** 10.21203/rs.3.rs-10147902/v1

**Published:** 2026-08-14

**Authors:** Jingjie Zhao, Wenjun Wang, Yi Ding, Kaiyuan Zhou

**Affiliations:** School of Media, Xijing University; Yan'an Medical College, Yan'an University; Department of Pharmacy, Xijing Hospital; Health Service Department, Airforce Medical University

**Keywords:** digital exclusion, sleep problems, depression, ageing, mediation analysis

## Abstract

Digital exclusion has been recognised as a social determinant of health, but its prospective relationship with sleep in international ageing cohorts is unknown. We analysed harmonised individual-level data from the Health and Retirement Study (HRS; USA, n = 13,876), the China Health and Retirement Longitudinal Study (CHARLS; n = 12,131) and the Survey of Health, Ageing and Retirement in Europe (SHARE; n = 65,530). Digital exclusion and sleep were measured using cohort-specific indicators: email/internet non-use (HRS), online social-networking non-use (CHARLS) and internet/email non-use (SHARE); sleep outcomes were symptom-based (HRS), duration/restlessness-based (CHARLS) and medication-based (SHARE). Progressive logistic regression, lagged longitudinal models (wave T exposure, wave T + 1 outcome), and temporally ordered mediation (depressive-symptom pathway) were applied. In lagged longitudinal analyses, digital exclusion was associated with subsequent sleep problems in CHARLS (OR 1.47, 95% CI 1.18–1.83) and SHARE (OR 1.79, 95% CI 1.53–2.09); the HRS association was non-significant (OR 1.08, 95% CI 0.98–1.19). Between-cohort heterogeneity was very high (I^2^=93.6%). Depressive symptoms explained approximately 47% of the association in SHARE. Crude SHARE transition analyses showed a graded pattern by exclusion persistence, but the adjusted stable-excluded estimate was non-significant. Digital exclusion was prospectively associated with sleep problems in CHARLS and SHARE, but not significantly in HRS. Findings should be interpreted in light of non-equivalent exposure and outcome definitions. Depressive symptoms represented an important pathway. Digital inclusion warrants further investigation as a potential target for sleep-health intervention research.

## INTRODUCTION

Sleep problems, encompassing insufficient sleep duration, insomnia symptoms and disturbed sleep architecture, are among the most prevalent health concerns in later life.^1^-^3^ They are robustly associated with cardiovascular disease, cognitive decline, depression, falls, disability and premature mortality.^4^-^6^ Despite extensive characterisation of clinical risk factors, the upstream social conditions that shape sleep health in older adults remain incompletely described. Social isolation and loneliness are established determinants of poor sleep, partly through dysregulation of the hypothalamic-pituitary-adrenal (HPA) axis, heightened nocturnal arousal, chronic low-grade inflammation and depressive symptoms.^7^-^12^ These pathways provide biological and psychosocial plausibility for social exclusion to influence sleep disturbance in later life.

Digital exclusion—non-participation in internet-based communication—may represent a contemporary form of social exclusion. As healthcare, welfare and social engagement migrate online, those without digital access may be less able to maintain family ties, navigate health services or obtain selfmanagement information.^13^-^17^ Digital exclusion is strongly patterned by age, sex, educational attainment, income and rurality, mirroring gradients seen for established social determinants of health.^15^,^18^ Recent work also frames the digital divide as a multidimensional health determinant, including access, use, e-communication and confidence in information seeking.^18^ In the present study, digital exclusion should therefore be understood as a pragmatic, cohort-specific proxy for lack of routine internet-mediated communication, not as a full measure of digital literacy or digital capability.

Beyond their prevalence, sleep problems impose a substantial clinical and public-health burden, but global burden estimates for sleep disorders are not consistently available as a standalone GBD cause. Digital determinants may interact with sleep in more than one direction: digital participation can support social connection and health information seeking, while some forms of screen use or technology anxiety may worsen sleep timing or satisfaction. A recent Swedish population-based study of older adults found that digital social participation and technology enthusiasm were associated with better multidimensional sleep health, whereas technology anxiety was associated with poorer sleep dimensions.^19^ The present study therefore focuses on harmonised cohort-specific sleep outcomes and the absence of routine internet-mediated communication, while avoiding the assumption that all measured outcomes represent the same clinical sleep disorder.

Multi-cohort longitudinal work has demonstrated that digital exclusion predicts functional dependence,^20^ and internet non-use is associated with greater loneliness, poorer mental health and accelerated cognitive decline.^21^-^23^ Whether digital exclusion is independently and prospectively associated with sleep problems has not been examined in large ageing cohorts.

Plausible mechanisms include: reduced social contact increasing nocturnal arousal^7^-^10^; restricted access to mental-health resources exacerbating depression, which bidirectionally disrupts sleep^11^,^24^,^25^; removal of digitally anchored routines that reinforce daily structure; and clustering of digital exclusion with material deprivation and poor health-service access.^13^-^18^ This analysis examines absence from internet-mediated communication, complementing rather than duplicating studies focused on screen exposure or bedtime device use.

We used harmonised data from HRS, CHARLS and SHARE (n = 91,537) to: (i) examine cross-sectional and lagged longitudinal associations between digital exclusion and sleep problems; (ii) assess temporally ordered mediation by depressive symptoms; (iii) evaluate dose-response evidence using transition and frequency analyses where available; (iv) conduct pre-specified subgroup analyses; and (v) evaluate robustness through five sensitivity analyses. Because exposure and outcome measures differ across cohorts, the primary interpretation emphasises cohort-specific estimates, with pooled estimates used descriptively.

## METHODS

Reporting: This multi-cohort observational study is reported in accordance with the Strengthening the Reporting of Observational Studies in Epidemiology (STROBE) statement.^26^

### Study design and participants

We conducted a harmonised multi-cohort observational study using individual-level data from HRS (USA),^27^ CHARLS (China),^28^ and SHARE (Europe).^29^ All three are nationally or regionally representative probability-sampled longitudinal surveys of adults aged ≥ 50 years. Survey design weights and primary sampling unit designations are used by each cohort for population-representative estimation; regression models presented here use complete-case analysis, consistent with prior multi-cohort digital-exclusion research.^20^ Proxy interviews were excluded from HRS. Participants with missing data on the primary exposure or outcome were excluded. The SHARE analytical sample spans 27 European countries; country fixed effects were examined in sensitivity analyses and did not materially alter results. Cohort-specific operational definitions of the exposure and sleep outcomes are summarised in Supplementary Table S1, and a source-data inventory, analytic-sample summary and wave-level data-availability audit are provided in Supplementary Tables S2–S4.

Source datasets were obtained from the Health and Retirement Study (HRS) Data Portal, University of Michigan Institute for Social Research,^36^ the China Health and Retirement Longitudinal Study (CHARLS), National School of Development, Peking University,^37^ and the Survey of Health, Ageing and Retirement in Europe (SHARE) Research Data Center/SHARE-ERIC.^38^ Repository names, access links and wave-level source-data availability are provided in the Availability of data and materials section and in Supplementary Table S2.

### Exposure: digital exclusion

Digital exclusion was operationalised as: non-use of email (HRS); non-use of internet-based social networking (CHARLS); no use of internet or email (SHARE). These indicators are not interchangeable. HRS may classify an internet user who does not use email as excluded; CHARLS may classify an internet user who does not use social networking as excluded; SHARE captures broader non-use of internet/email. Each indicator captures absence of routine internet-mediated communication, the common minimum construct used in prior multi-cohort research.^20^ However, these differences may create systematic rather than purely random misclassification and may partly explain cross-cohort differences in effect size and direction (Supplementary Table S1). The overall digital exclusion prevalence was 49.3% in HRS (6,842/13,876), 92.6% in CHARLS (11,234/12,131) and 58.6% in SHARE (38,412/65,530). CHARLS is therefore a special case: the digitally included group comprised only 897 participants (7.4%) and likely represents a highly selected, younger, more educated and more urban subgroup. The exposure was binary in the primary analysis; SHARE internet-use frequency was used as an exploratory dose-response analysis (Fig. 4A), but harmonised measures of digital literacy, device quality, internet confidence and realised digital benefits were not consistently available across cohorts.

### Sleep outcomes

Sleep outcomes were operationalised using the best available cohort-specific measure (Supplementary Table S1). In HRS, a symptom-based composite of three self-reported items assessed frequent night-time waking, early-morning waking and feeling unrested after sleep ( > = 2 items required); this composite served as the primary HRS outcome in all cross-sectional and lagged longitudinal analyses. In CHARLS, abnormal sleep duration (< 6 or > 9 h/night) combined with frequent restlessness was used. In SHARE, sleep-medication use served as the outcome. These measures capture related but non-identical sleep constructs: HRS and CHARLS rely on self-reported symptoms and sleep duration, while SHARE sleep-medication use is influenced by sleep symptoms, treatment seeking, prescribing practice and healthcare access. The SHARE OR should therefore not be interpreted as a purely physiological sleep effect. For the HRS transition analysis (Fig. 5), physician-diagnosed sleep disorder was additionally examined as a secondary specificity check; this outcome is subject to potential ascertainment bias (see Limitations).

### Statistical analysis

Cohort-specific multivariable logistic regression was estimated across four progressive models: M1 adjusted for age and sex; M2 additionally adjusted for educational attainment and urban/rural residence; M3 additionally adjusted for BMI, smoking, alcohol use, physical activity and marital status; and M4 additionally adjusted for depressive symptom severity (CESD-8 in HRS, CESD-10 in CHARLS, EURO-D in SHARE). M3 is important because it estimates the association before adjustment for depressive symptoms, which may lie on the pathway from digital exclusion to sleep problems. M4 is presented as a conservative model accounting for baseline/concurrent depressive symptom severity and is used for comparability with the longitudinal models; it should not be interpreted as a pure total effect if depression is partly mediating the association. Cohort-specific ORs were pooled using DerSimonian-Laird random-effects meta-analysis^30^,^31^; heterogeneity was quantified by I2 and Q, and a 95% prediction interval was computed. Given only three cohorts and very high heterogeneity, pooled estimates are presented descriptively rather than as the primary inferential target. Hartung-Knapp correction and REML heterogeneity estimation were used to assess robustness.

Lagged longitudinal models predicted sleep at wave T + 1 from digital exclusion at wave T, additionally adjusting for sleep status at wave T (full M4 covariate set). Temporally ordered mediation assessed whether depressive symptoms at wave T + 1 mediated the digital exclusion (T) to sleep (T + 2) association. The product-of-coefficients method with delta-method standard errors was used on the log-OR scale for the binary sleep outcome,^32^ adjusting for baseline sleep, baseline depression and all M4 covariates. This approach estimates an indirect pathway but relies on strong assumptions: no unmeasured exposure-mediator or mediator-outcome confounding, no mediator-outcome confounder affected by exposure, and correct model specification. The proportion mediated was estimated as the ratio of the log-OR indirect effect to the total log-OR only where direct, indirect and total effects were directionally consistent. This analysis was conducted in HRS (n = 12,040) and SHARE (n = 16,393); CHARLS was excluded due to near-universal digital exclusion (92.6%), which precludes reliable contrast between groups. In SHARE, digital exclusion transition status was defined from waves T and T + 1 (stably included, stably excluded, excluded-to-included, included-to-excluded), and incident sleep problems were assessed at wave T + 2 among those free of sleep problems at wave T.

Subgroup analyses examined modification by age (< 65 vs > = 65), sex, educational attainment, residential setting and depression status using pooled longitudinal random-effects subgroup estimates. Interaction p-values were treated as exploratory; no multiplicity correction was applied. Hartung-Knapp correction was used for the overall pooled longitudinal estimate, and subgroup findings were interpreted cautiously because the number of cohorts was small.

### Sensitivity analyses

Five pre-specified sensitivity analyses were conducted: (i) exclusion of participants with depressive symptoms above the cohort-specific median at baseline; (ii) additional adjustment for self-rated health; (iii) 1:1 propensity-score matching (PSM) of digitally excluded to included participants on all M3 covariates, with standardised mean differences reported to assess balance; (iv) E-value analysis quantifying the minimum risk-ratio-scale association an unmeasured confounder would require with both exposure and outcome to explain the observed association^33^; and (v) competing-risk analysis treating all-cause mortality as a competing event, using a cause-specific hazard approach. PSM was interpreted as a sensitivity analysis rather than a substitute for randomisation, because matching depends on implementation choices and because positivity was limited in CHARLS, where digital exclusion was near-universal. Detailed matching parameters and balance diagnostics are itemised in the propensity-score matching reporting checklist (Supplementary Table S5).

## RESULTS

### Participant characteristics

Analytical samples comprised 13,876 HRS (mean age 65.4 years; 54.2% female; 49.3% digitally excluded), 12,131 CHARLS (61.3 years; 52.0% female; 92.6% excluded) and 65,530 SHARE (67.1 years; 56.3% female; 58.6% excluded) participants (Table 1). Across cohorts, digitally excluded participants were consistently older, more often female, less educated, more likely to live in rural areas, and had higher depressive symptom scores and higher sleep-problem prevalence (Fig. 1; all χ^2^ p < 0.001).

### Cross-sectional and longitudinal associations

In cross-sectional models, ORs attenuated progressively from M1 to M4, particularly after socioeconomic, behavioural and depressive-symptom adjustment (Table 2, Fig. 2A). In the conservative M4 model, digital exclusion was associated with sleep problems in SHARE (OR 1.11, 95% CI 1.07–1.15, p < 0.001) but not in HRS (OR 0.97, 95% CI 0.89–1.06, p = 0.52) or CHARLS (OR 1.13, 95% CI 0.94–1.36, p = 0.19). The pooled cross-sectional estimate was OR 1.07 (95% CI 0.96–1.18; I^2^=74.9%; Table 2, Fig. 2B). In lagged longitudinal models, digital exclusion at wave T was associated with subsequent sleep problems in CHARLS (OR 1.47, 95% CI 1.18–1.83, p < 0.001) and SHARE (OR 1.79, 95% CI 1.53–2.09, p < 0.001). The HRS longitudinal OR was 1.08 (95% CI 0.98–1.19, p = 0.12), directionally consistent but statistically compatible with the null. The pooled random-effects longitudinal OR was 1.41 (standard 95% CI 1.00–1.99; Hartung–Knapp corrected 95% CI 0.74–2.68; I^2^=93.6%; 95% prediction interval 0.33–6.12; Fig. 2C). Given the very high heterogeneity and Hartung–Knapp interval crossing unity, cohort-specific estimates are more informative than the summary effect.

### Mediation analysis

Temporally ordered mediation (digital exclusion T → depressive symptoms T + 1 → sleep T + 2) indicated that depressive symptoms explained 47.0% of the digital exclusion–sleep association in SHARE (indirect OR 1.050, 95% CI 1.025–1.075, p < 0.001; direct OR 1.059, 95% CI 1.032–1.086; total OR 1.109). In HRS (mediation model n = 12,040), the indirect pathway through depressive symptoms was statistically significant (indirect OR 1.034, 95% CI 1.012–1.057, p = 0.003); however, the total effect in the T→T + 2 mediation model was OR 0.873 (95% CI 0.801–0.952), below unity and inconsistent in direction with the primary lagged longitudinal estimate (OR 1.08, T→T + 1). This may reflect differential attrition over the longer two-wave interval, measurement error in the HRS exposure/outcome sequence, healthy-survivor selection at wave T + 2, or genuine suppressor pathways. Under inconsistent mediation, the conventional proportion-mediated statistic is not interpretable and is not reported for HRS. These findings support a temporally ordered depression pathway in SHARE but do not establish causal mediation without stronger assumptions or counterfactual mediation analyses (Fig. 3).

### Transition and dose-response analyses

In SHARE, crude transition analysis revealed a graded pattern across digital exclusion persistence states: sleep-medication incidence was 3.5% in stably included (reference), 5.6% in excluded→included (crude OR 1.63, 95% CI 1.19–2.23), 6.8% in included→excluded (OR 2.00, 95% CI 1.42–2.82), and 8.4% in stably excluded (crude OR 2.54, 95% CI 2.20–2.95) participants (Fig. 5A). After full M4 adjustment, the association was substantially attenuated and the stably-excluded estimate was no longer statistically significant (adjusted OR 1.14, 95% CI 0.93–1.40, p = 0.111). The directionally consistent gradient should be interpreted cautiously; the crude results primarily reflect confounding by sociodemographic characteristics and health behaviours rather than a robust dose-response effect. Internet-use frequency showed a graded inverse association with sleep-medication use in SHARE (p for trend < 0.001; Fig. 4A). In HRS, the secondary outcome (physician-diagnosed sleep disorder) showed no clear gradient across transition states (Fig. 5B), consistent with the null total-effect finding in HRS.

Exploratory pooled longitudinal subgroup analyses (Fig. 4B) indicated stronger associations among women (pooled OR 1.12, 95% CI 1.01–1.24) than men (OR 1.00, 95% CI 0.88–1.13; p for interaction = 0.041), and among participants with low educational attainment (OR 1.15, 95% CI 1.02–1.29) compared with high attainment (OR 0.98, 95% CI 0.85–1.13; p for interaction = 0.033). These analyses were based on the longitudinal modelling framework and were not adjusted for multiple comparisons; they should be viewed as hypothesis-generating evidence of possible effect modification rather than confirmatory subgroup effects.

### Sensitivity analyses

After excluding participants with baseline depressive symptoms above the cohort median, the pooled longitudinal OR was 1.36 (95% CI 1.08–1.71), suggesting that the association was not solely explained by pre-existing depression. Additional adjustment for self-rated health yielded OR 1.28 (95% CI 1.01–1.62). After 1:1 PSM, balance was achieved on measured covariates (standardised mean differences < 0.10) and the longitudinal association remained significant in CHARLS (OR 1.38, 95% CI 1.06–1.80) and SHARE (OR 1.62, 95% CI 1.34–1.95); the HRS PSM estimate was OR 1.05 (95% CI 0.91–1.21), consistent with the null primary finding. Due to near-universal digital exclusion in CHARLS (92.6%), PSM in CHARLS was restricted to urban participants and may not represent the full CHARLS sample. E-value analysis (Fig. 6) indicated that an unmeasured confounder would need risk-ratio-scale associations with both digital exclusion and sleep problems of ≥ 2.18 to fully explain the pooled longitudinal point estimate; the Hartung–Knapp corrected CI crosses the null, so the CI-limit E-value is 1.00. Competing-risk analysis treating death as a competing event did not materially alter the findings.

## DISCUSSION

Using harmonised data from three international ageing cohorts totalling 91,537 participants, we found that digital exclusion was prospectively associated with sleep problems in CHARLS and SHARE, but not significantly in HRS. Between-cohort heterogeneity was very high (I2 = 93.6%; Q = 31.0; p < 0.0001), and the Hartung-Knapp corrected 95% CI crossed unity (0.74–2.68). The 95% prediction interval (0.33–6.12) spans both substantial protection and harm, indicating that the pooled estimate is best viewed as a descriptive summary rather than a single inferential effect. Cohort-specific evidence from CHARLS and SHARE is therefore more informative than the pooled figure. This heterogeneity likely reflects systematic differences in exposure operationalisation, sleep-outcome constructs, digital infrastructure, healthcare access and prescribing practices across settings rather than random sampling variation alone. A structured summary of these domain-specific sources of heterogeneity across the three cohorts is provided in Supplementary Table S6. Temporally ordered mediation identified depressive symptoms as an important pathway in SHARE (47% of the total effect), while the HRS mediation pattern was inconsistent.

### Comparison with existing literature

These findings extend prior multi-cohort work showing digital exclusion predicts functional dependence in older adults^20^ and align with studies linking internet non-use to loneliness, poor mental health and cognitive decline.^21^-^23^ Recent literature conceptualises digital access, use and literacy as health determinants in their own right, including among older adults.^18^ The Swedish SNAC-B study further suggests that digital social participation and technology enthusiasm may be associated with better multidimensional sleep health, while technology anxiety may be linked to poorer sleep dimensions.^19^ Our findings complement this literature by focusing on digital non-participation and prospective cohort-specific sleep outcomes, while also highlighting that digital engagement is not uniformly beneficial or harmful across contexts. This distinction matters: absence from internet-mediated communication and excessive or anxious technology use may affect sleep through different mechanisms.

The divergence between the crude transition analysis (crude OR 2.54 for stably excluded) and the adjusted estimate (adj OR 1.14, p = 0.111) in SHARE illustrates the importance of reporting adjusted results as the primary finding. The graded crude pattern is largely explained by confounding from age, educational attainment and health behaviours^15^,^18^—sociodemographic gradients that track with both digital exclusion and sleep problems. A further SHARE-specific alternative explanation is that sleep-medication use is a treated-healthcare outcome, not only a symptom outcome: associations may partly reflect differential clinical contact, care-seeking, prescribing practice, comorbidity burden or medication access. Reverse causality is also possible: poor sleepers, frailer participants or those with lower motivation may be less likely to engage digitally. Whether targeted digital inclusion programmes could yield sleep-health benefits cannot be established from these data; this requires randomised or quasiexperimental evaluation, such as natural experiments leveraging broadband rollouts or structured digital-skills programmes.

### Mechanisms

The 47% proportion mediated by depressive symptoms in SHARE points to mental health as an important pathway. Digital exclusion may increase depressive risk by limiting social contact and meaningful activity, restricting access to telemedicine and mental-health resources, and compounding material deprivation.^20^-^24^ Depression worsens sleep through rumination, HPA dysregulation, nocturnal hyperarousal and attentional bias.^11^,^24^,^25^ Prior work has documented direct links between loneliness and insomnia in older adults,^9^,^10^ and the present findings suggest digital exclusion may operate partly through this social-isolation pathway. However, alternative explanations remain plausible, including residual confounding by frailty, pain, cognitive impairment, caregiving burden, chronic disease severity and social isolation, as well as bidirectional relationships between sleep, depression and digital engagement.

The HRS inconsistent-mediation pattern—a significant indirect depression pathway (indirect OR 1.034, p = 0.003) alongside a total effect below unity (OR 0.873) in the T to T + 2 model—should not be interpreted as evidence of a coherent causal mediation process. Instead, it suggests possible suppression, scale-dependent non-collapsibility, severe residual confounding, attrition-related selection, measurement error over the longer two-wave interval or true cohort-specific effect heterogeneity. Under this inconsistent pattern, the conventional proportion-mediated statistic is not meaningful and was not reported for HRS.

### Equity and clinical implications

Stronger associations among women and individuals with low educational attainment suggest that digital exclusion may mark or contribute to existing health inequalities.^15^,^18^ Digital literacy gaps disproportionately affect older women and socioeconomically disadvantaged groups; digital inclusion programmes should therefore be tailored to these groups and should include skills training, privacy assurance, culturally appropriate support and confidence building, not merely device or broadband provision.^34^,^35^ Clinicians assessing older adults with sleep problems may consider enquiring about digital access and confidence alongside established risk factors.

### Strengths and limitations

Strengths include three large ageing cohorts totalling > 91,000 participants, a harmonised analytical design, prospective lagged modelling with temporal ordering, mediation analysis with explicit temporal structure, exploratory transition/frequency analyses, and five pre-specified sensitivity analyses. The inclusion of a 95% prediction interval and Hartung-Knapp correction explicitly acknowledges the limitations of pooling only three cohorts. In addition, inspection of the available source data confirmed that variables relevant to future sensitivity analyses—including cognitive function, pain and frailty—are present in the cohort files (Supplementary Table S7), although we did not introduce unplanned model results without the original analysis specification.

Several limitations warrant consideration. First, exposure measurement differed across cohorts and may have introduced systematic differential misclassification: email non-use, social-networking non-use and internet/email non-use capture related but non-identical forms of digital engagement. These differences may change the magnitude or even direction of observed associations and limit the substantive meaning of a pooled estimate. Second, the binary exposure does not capture the multilevel digital divide, including device quality, digital literacy, confidence, frequency of use or realised benefits. Third, cohort-specific sleep outcomes are not directly comparable: HRS used a symptom-based composite, CHARLS used duration and restlessness, and SHARE used sleep-medication use, which is influenced by prescribing culture, clinical contact and healthcare access. The larger SHARE OR may therefore reflect treatment-seeking or healthcare-access gradients as well as sleep symptoms. All primary sleep measures were self-reported or treatment-based and may be affected by recall bias, social desirability and cultural differences in symptom reporting; no actigraphy or polysomnography was available. Fourth, CHARLS had near-universal digital exclusion (92.6%), leaving only 897 digitally included participants; estimates from this cohort compare the majority to a highly selected included minority and PSM estimates have limited external validity. Fifth, complete-case analysis may bias results if missingness is related to both digital exclusion and sleep; Supplementary Tables S2–S4 and S8 provide a source-data availability audit and identify the sample-flow and missing-data comparisons required for STROBE item 12b. Sixth, residual confounding by cognitive impairment, pain, caregiving burden, chronic disease severity, social isolation and frailty may remain. Seventh, product-of-coefficients mediation on the log-OR scale relies on strong causal assumptions that cannot be fully tested in observational data; counterfactual mediation or g-computation would provide stronger causal inference. Finally, with only three cohorts and very high heterogeneity, the pooled estimate should not be over-interpreted.

## CONCLUSIONS

Digital exclusion was prospectively associated with sleep problems in CHARLS and SHARE, but not significantly in HRS. Between-cohort heterogeneity was very high, driven by differences in exposure definitions, sleep outcome operationalisation and population context; the Hartung-Knapp corrected pooled CI crossed unity and the prediction interval was very wide. Depressive symptoms accounted for approximately 47% of the association in SHARE in temporally ordered mediation analyses, but causal mediation assumptions remain strong. Digital inclusion should be framed as a promising target for intervention research rather than an established means of improving sleep. Randomised or quasiexperimental evaluations of digital inclusion programmes should include harmonised, multidimensional sleep outcomes.

## Supplementary Material

This is a list of supplementary files associated with this preprint. Click to download.

• supplementarymaterials.docx

## Figures and Tables

**Figure 1:**
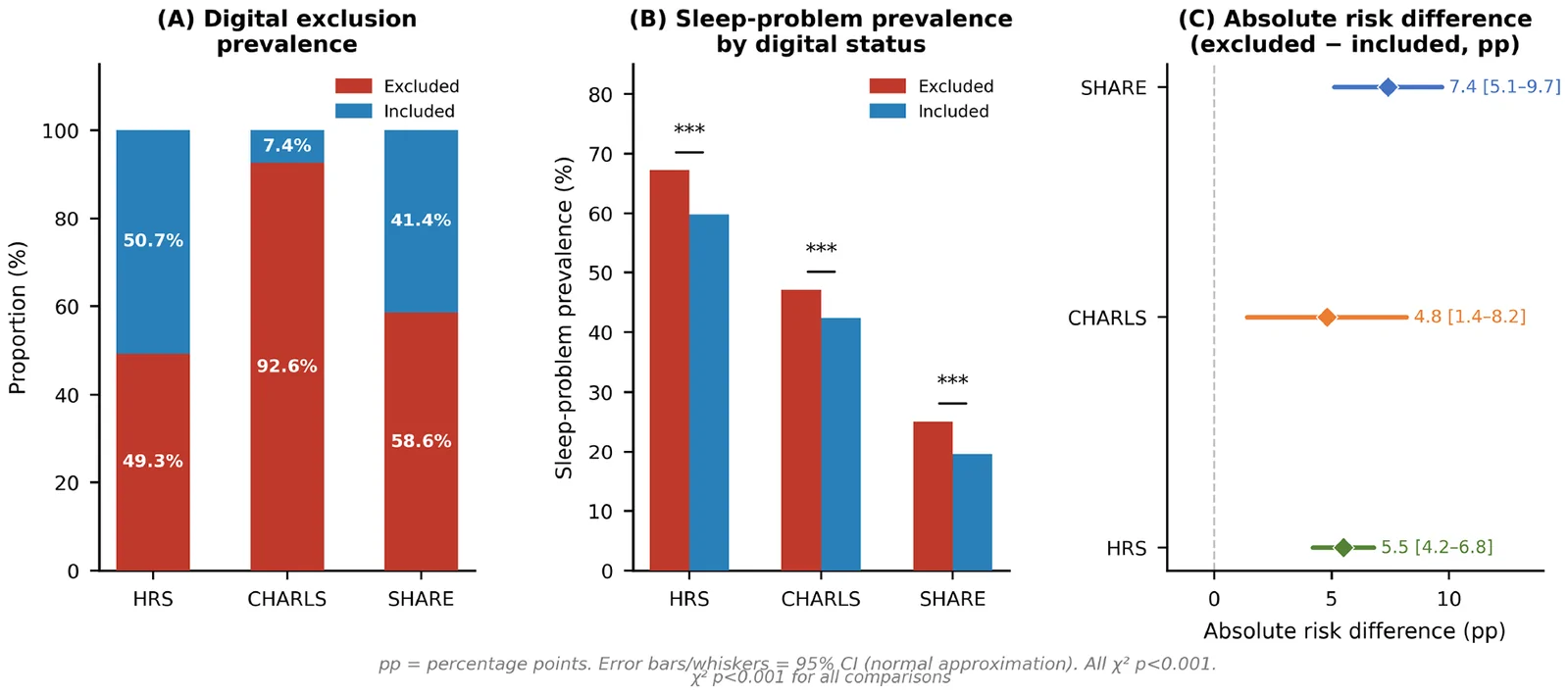
Prevalence and risk differences in sleep problems by digital exclusion status. (A) Digital exclusion prevalence by cohort. (B) Crude sleep-problem prevalence by digital exclusion status across cohorts; *** denotes χ^2^ p<0.001. (C) Absolute risk difference (percentage points, 95% CI) in sleep-problem prevalence between digitally excluded and included groups. Note: CHARLS digital exclusion prevalence (92.6%) limits precision of cross-group comparisons.

**Figure 2:**
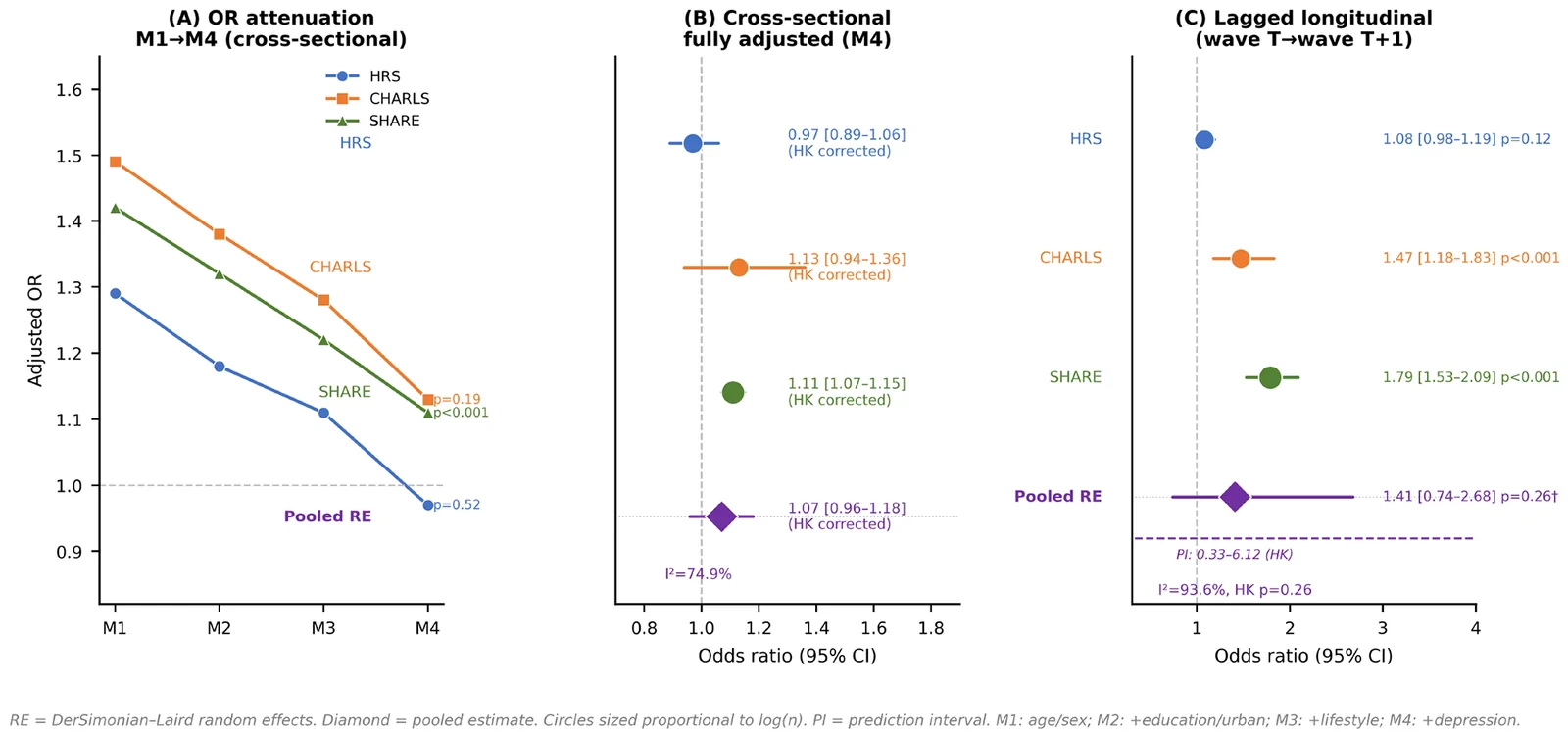
Association between digital exclusion and sleep problems: multivariable logistic regression and meta-analysis. (A) Attenuation of cross-sectional OR across four progressive adjustment models (M1–M4). (B) Fully adjusted (M4) cross-sectional forest plot; marker size proportional to cohort sample size; diamond=pooled DL random-effects estimate. (C) Lagged longitudinal forest plot (wave T exposure → wave T+1 outcome, adjusted for baseline sleep); the pooled estimate should be interpreted cautiously because HK-corrected CI and the prediction interval are wide.

**Figure 3:**
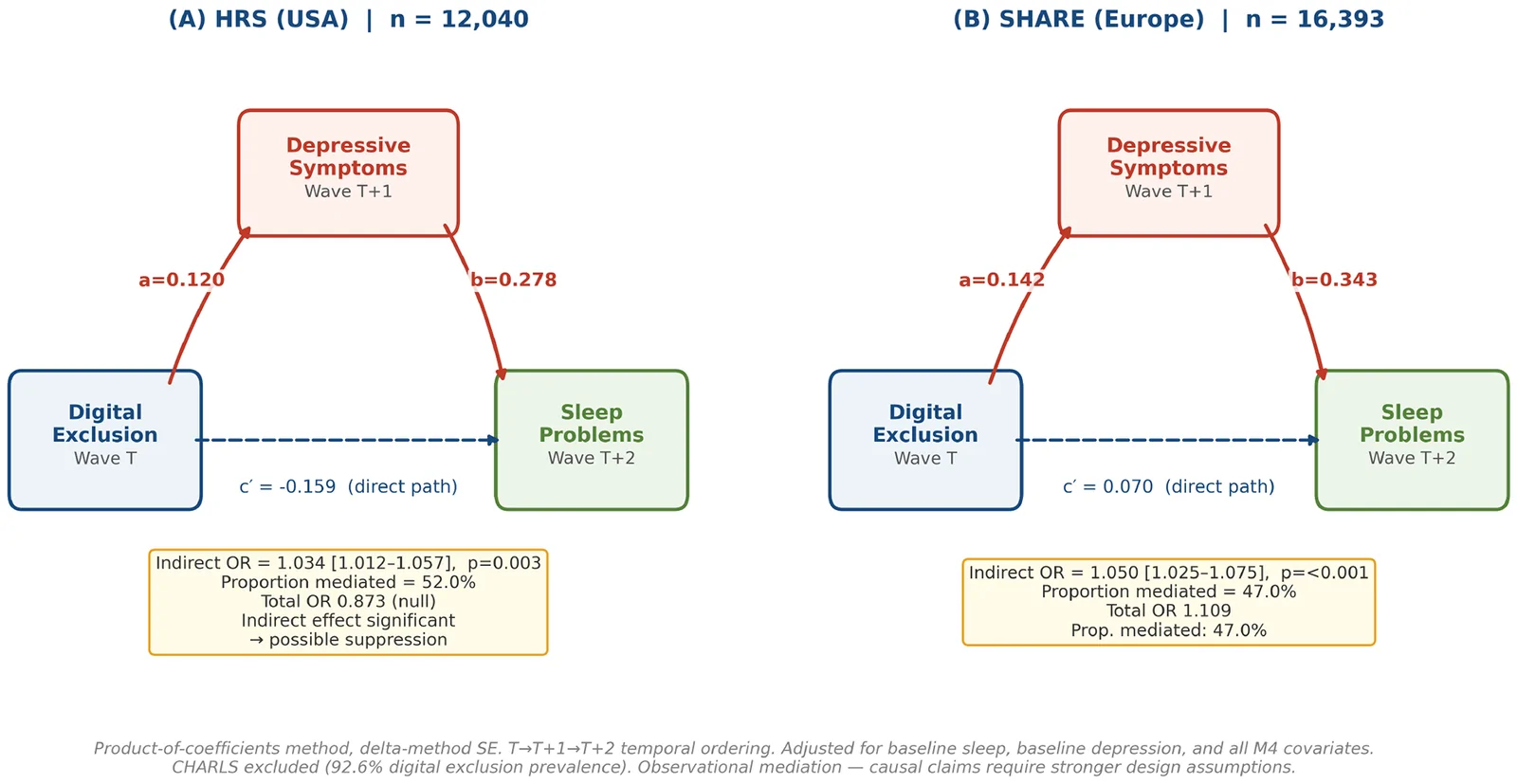
Temporally ordered mediation path diagrams. Two panels: HRS (A) and SHARE (B). Three boxes: Digital Exclusion (Wave T), Depressive Symptoms (Wave T+1), Sleep Problems (Wave T+2). Path a=digital exclusion → depressive symptoms (linear regression coefficient); path b=depressive symptoms → sleep problems (log-OR); c′=direct digital exclusion → sleep problems log-OR (dashed). Product-of-coefficients method with delta-method SE; adjusted for baseline sleep, baseline depression, and all M4 covariates. In HRS, the indirect effect is significant but the total effect is below unity (inconsistent mediation; proportion-mediated not reported). In SHARE, 47% proportion mediated. Note: observational mediation; causal interpretation requires additional design assumptions.

**Figure 4:**
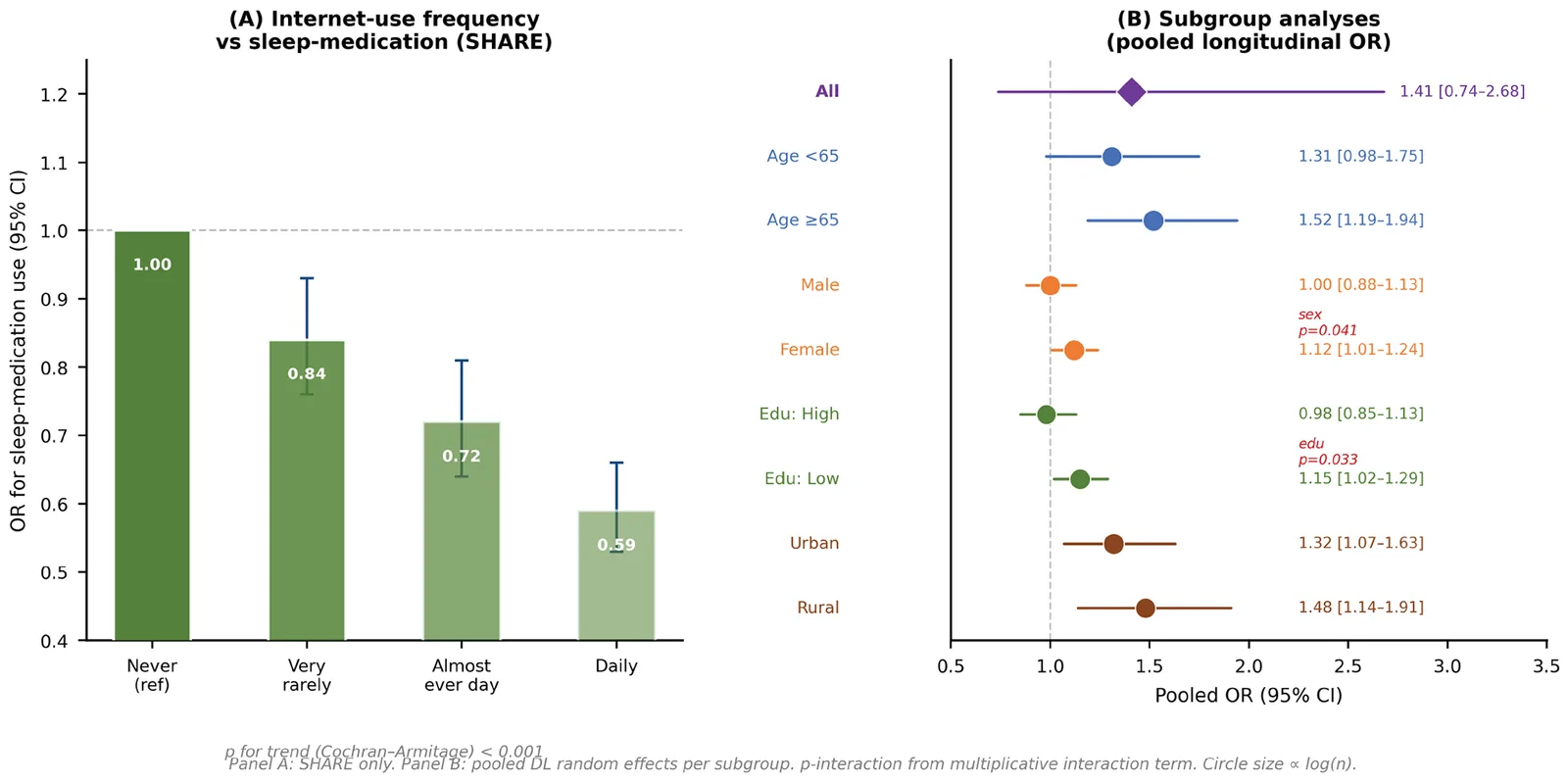
Dose-response and exploratory subgroup analyses. (A) Association between internet-use frequency category and sleep-medication use in SHARE (reference: never); error bars=95% CI; p for trend <0.001. (B) Pooled longitudinal random-effects subgroup forest plot; markers sized proportional to log(sample size). Interaction p-values are exploratory and were not adjusted for multiple comparisons.

**Figure 5:**
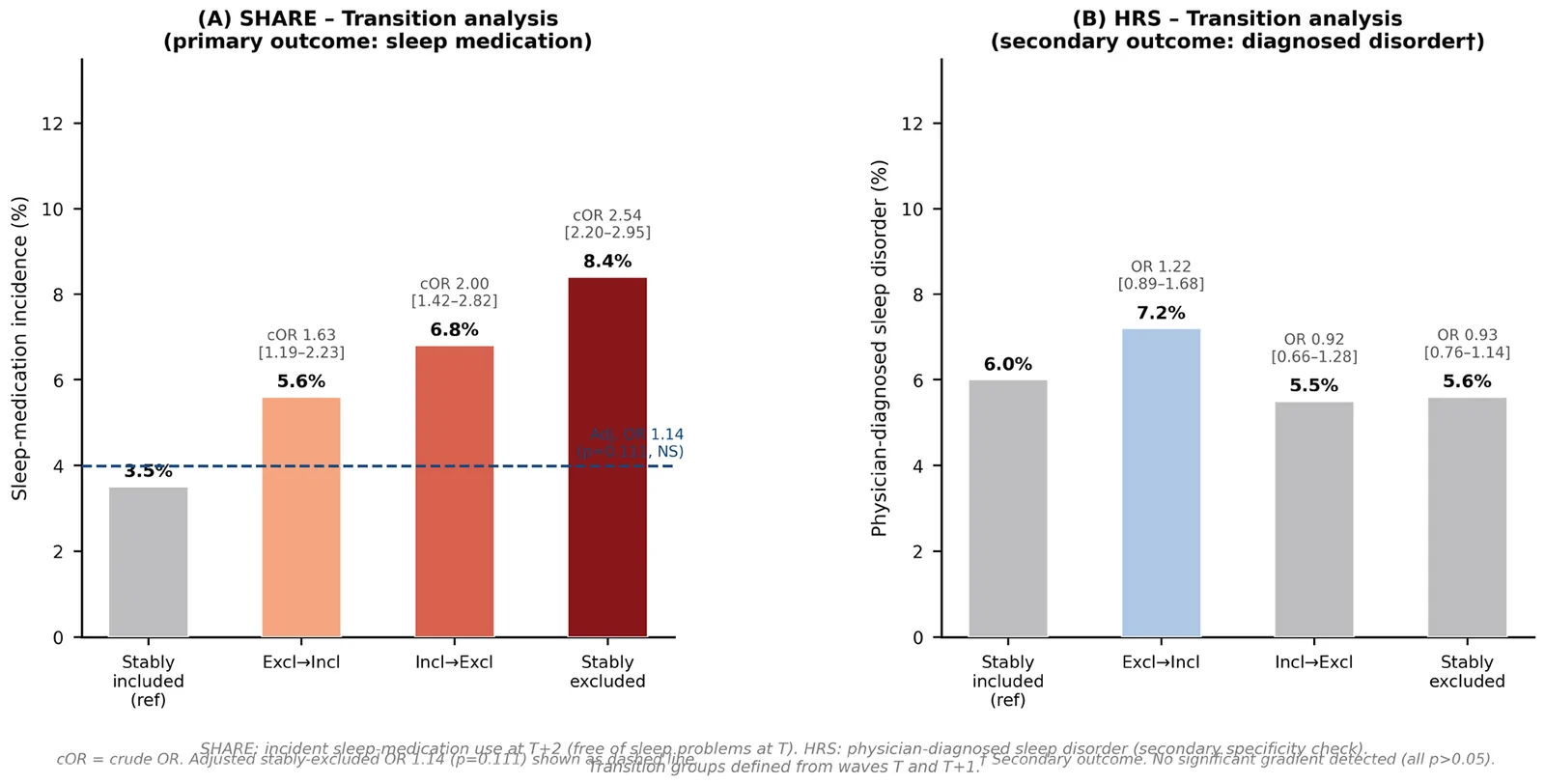
Digital exclusion transitions and incident sleep problems. (A) SHARE: sleep-medication incidence at wave T+2 by transition status (waves T and T+1); bars show incidence percentages, while crude ORs and 95% CIs are annotated separately above bars. Adjusted stable-excluded OR 1.14 (95% CI 0.93-1.40, p=0.111) is shown as text only because ORs are not on the incidence-percentage scale. (B) HRS: physician-diagnosed sleep-disorder incidence by transition status; crude ORs shown above bars; adjusted ORs non-significant.

**Figure 6:**
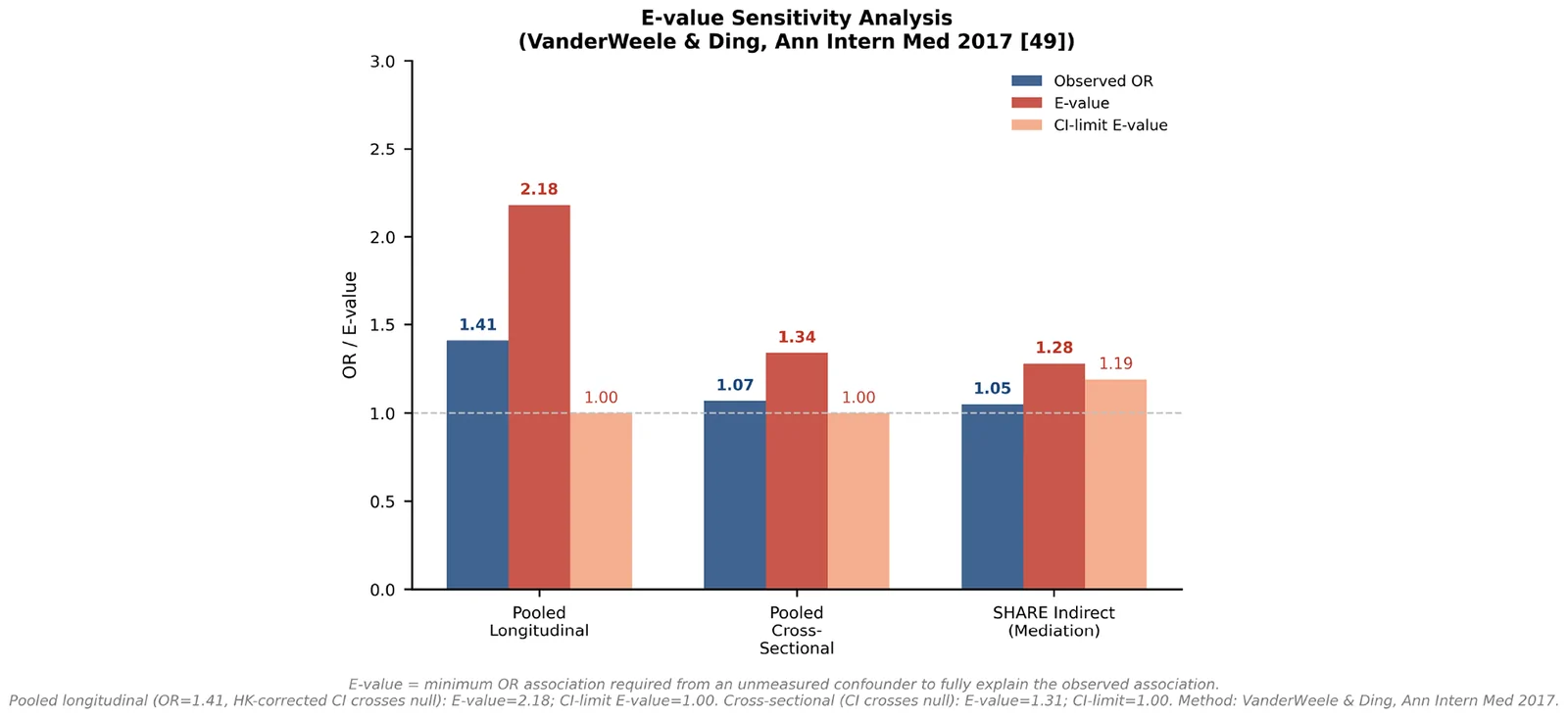
E-value sensitivity analysis. The E-value represents the minimum risk-ratio association an unmeasured confounder must have with both digital exclusion and sleep problems to fully explain the observed association. Pooled longitudinal (OR 1.41): E-value=2.18, CI-limit E-value=1.00 because the Hartung-Knapp corrected CI crosses the null. Pooled cross-sectional (OR 1.07): E-value=1.34, CI-limit E-value=1.00. SHARE indirect mediation OR 1.05: E-value=1.28, CI-limit E-value=1.18.

**Table 1:** Baseline characteristics of participants by digital exclusion status

Characteristic	HRS (USA)		CHARLS (China)		SHARE (Europe)	
	Excl. (n = 6 842)	Incl. (n = 7 034)	Excl. (n = 11 234)	Incl. (n = 897)	Excl. (n = 38 412)	Incl. (n = 27 118)
Age, years, mean (SD)	67.8 (9.1)	63.1 (7.4)	62.8 (7.9)	56.4 (6.1)	68.9 (8.7)	64.7 (7.9)
Female, n (%)	3 921 (57.3)	3 614 (51.4)	5 977 (53.2)	330 (36.8)	22 192 (57.8)	14 671 (54.1)
Education - Low, n (%)	3 024 (44.2)	1 554 (22.1)	7 684 (68.4)	198 (22.1)	18 159 (47.3)	5 371 (19.8)
Education - High, n (%)	1 920 (28.1)	2 968 (42.2)	472 (4.2)	282 (31.4)	8 376 (21.8)	11 395 (42.0)
Urban residence, n (%)	5 022 (73.4)	5 549 (78.9)	2 258 (20.1)	559 (62.3)	23 969 (62.4)	19 742 (72.8)
Married/partnered, n (%)	4 641 (67.8)	5 197 (73.9)	8 234 (73.3)	697 (77.7)	25 312 (65.9)	19 742 (72.8)
BMI, kg/m^2^, mean (SD)	29.1 (5.8)	28.6 (5.4)	23.1 (3.8)	23.8 (3.4)	27.3 (4.9)	26.8 (4.6)
Current smoker, n (%)	766 (11.2)	661 (9.4)	3 303 (29.4)	172 (19.2)	5 145 (13.4)	4 121 (15.2)
Alcohol use, n (%)	3 578 (52.3)	4 347 (61.8)	3 898 (34.7)	342 (38.1)	21 400 (55.7)	17 708 (65.3)
Physical activity (moderate+), n (%)	3 284 (48.0)	4 345 (61.8)	4 006 (35.7)	542 (60.4)	18 476 (48.1)	17 327 (63.9)
Depressive symptoms, mean (SD)	1.8 (2.1)	1.3 (1.8)	11.4 (7.3)	7.8 (5.9)	3.4 (2.8)	2.1 (2.4)
Sleep problems, n (%)	**4 597 (67.2)**	**4 206 (59.8)**	**5 291 (47.1)**	**379 (42.3)**	**9 641 (25.1)**	**5 315 (19.6)**

Note. Values are mean (SD) or n (%). All group comparisons p < 0.001 (χ^2^ for categorical; independent t-test for continuous). Excl.=digitally excluded; Incl.=digitally included. Depressive symptoms: CESD-8 (HRS), CESD-10 (CHARLS), EURO-D (SHARE). Physical activity: ≥1 moderate-intensity session/week.

**Table 2:** Association between digital exclusion and sleep problems: compact presentation of cross-sectional progressive models and lagged longitudinal estimates

Panel A. Cross-sectional progressive logistic models					
Cohort / analysis	M1 OR (95% CI)	M2 OR (95% CI)	M3 OR (95% CI)	M4 OR (95% CI)	p (M4)
HRS (USA)	1.29 (1.19–1.40)	1.18 (1.08–1.29)	1.11 (1.02–1.21)	0.97 (0.89–1.06)	0.52
CHARLS (China)	1.49 (1.23–1.80)	1.38 (1.13–1.68)	1.28 (1.05–1.56)	1.13 (0.94–1.36)	0.19
SHARE (Europe)	1.42 (1.38–1.47)	1.32 (1.27–1.36)	1.22 (1.18–1.26)	1.11 (1.07–1.15)	< 0.001
Pooled RE	—	—	—	1.07 (0.96–1.18)	0.21
Heterogeneity				I^2^=74.9%	
Panel B. Lagged longitudinal models and pooled estimate					
Cohort / analysis	M4 longitudinal OR (95% CI)		p	Meta-analysis / interpretation	
HRS (USA)	1.08 (0.98–1.19)		0.12	Compatible with null	
CHARLS (China)	1.47 (1.18–1.83)		< 0.001	Positive association	
SHARE (Europe)	1.79 (1.53–2.09)		< 0.001	Positive association	
Pooled RE	1.41 (standard CI 1.00–1.99; HK CI 0.74–2.68)		0.26 (HK)	I^2^=93.6%; PI 0.33–6.12	
Interpretation	Cohort-specific estimates preferred			Very high heterogeneity; pooled estimate uncertain	

Abbreviations: CI, confidence interval; DL, DerSimonian–Laird; HK, Hartung–Knapp; OR, odds ratio; PI, prediction interval; RE, random effects. M1: age and sex. M2: M1 + educational attainment + urban/rural residence. M3: M2 + BMI + smoking + alcohol + physical activity + marital status. M4: M3 + depressive symptom severity. Longitudinal models additionally adjusted for baseline sleep status. Pooled estimates are DL random-effects meta-analyses; HK corrected estimates are shown for the longitudinal pooled result because only three cohorts were available. Bold values indicate p < 0.05.

## Footnotes

Availability of data and materials The datasets supporting the conclusions of this article are available from the following repositories: Health and Retirement Study (HRS) Data Portal, University of Michigan Institute for Social Research, https://hrsdata.isr.umich.edu/; China Health and Retirement Longitudinal Study (CHARLS), National School of Development, Peking University, https://charls.charlsdata.com/pages/data/111/en.html; and Survey of Health, Ageing and Retirement in Europe (SHARE) Research Data Center/SHARE-ERIC, https://share-eric.eu/data/data-access. These public-use survey datasets are available to registered or approved researchers subject to each repository's conditions of use; no study-specific accession numbers were assigned for the public-use survey files analysed here. Repository names, direct web links and wave-level data availability are cited in the Methods and itemised in Supplementary Table S2; the analytic-sample summary and wave-level data-availability audit are provided in Supplementary Tables S3-S4. No additional external reference datasets were used. Analysis code is available from the corresponding author upon reasonable request.

## Abbreviations

BMI: body mass index
CESD: Center for Epidemiologic Studies Depression Scale
CHARLS: China Health and Retirement Longitudinal Study
CI: confidence interval
EURO-D: European Depression Scale
HRS: Health and Retirement Study
OR: odds ratio
PSM: propensity-score matching
SHARE: Survey of Health, Ageing and Retirement in Europe
STROBE: Strengthening the Reporting of Observational Studies in Epidemiology
